# The effect of thymus vulgaris extract and probiotic on growth performance, blood parameters, intestinal morphology, and litter quality of broiler chickens fed low-protein diets

**DOI:** 10.1016/j.psj.2024.104554

**Published:** 2024-11-22

**Authors:** Ali Golshahi, Mahmoud Shams Shargh, Behrouz Dastar, Enayat Rahmatnejad

**Affiliations:** a Department of Animal and Poultry Nutrition, Faculty of Animal Science, Gorgan University of Agricultural Sciences and Natural Resources, Gorgan, Iran; b Department of Animal Science, Faculty of Agriculture and Natural Resources, Persian Gulf University, Bushehr, 75169, Iran.

**Keywords:** Broiler chicken, Low-protein diet, Performance, Probiotic, Thyme

## Abstract

This study investigated the effects of Thymus vulgaris extract (**TVE**) and probiotic (Protexin) on Arian broiler chickens fed low protein diets over 42 days. The 2 × 3 factorial experiment involved two dietary crude protein (**CP**) levels and three supplementations, each with five replicates of 10 birds. The CP levels included a control group and a low-CP group with 5% reduced CP content. The supplementations were: no additive, probiotic at 0.1 g/kg diet, and TVE at 1 ml/L drinking water. GC-MS analysis of TVE identified linalool (28.54%), carvacrol (20.22%), and thymol (7.07%) as key bioactive compounds. Low-CP diets increased feed intake (**FI**) and feed conversion ratio (**FCR**) during the grower period (P > 0.05). Additives boosted FI and body weight gain (**BWG**) during the starter period, with probiotics having a stronger effect (P < 0.05). TVE improved protein efficiency ratio (**PER**) and energy efficiency ratio (**EER**), while both TVE and probiotics enhanced European production efficiency factor (**EPEF**) to levels like normal-CP diets (P < 0.05). Intestinal morphology was unaffected by treatments (P > 0.05). Low-CP diets reduced serum albumin levels (P < 0.05). TVE lowered serum cholesterol and triglycerides, while probiotic reduced triglycerides (P < 0.05). TVE also decreased alkaline phosphatase (**ALP**), and probiotics reduced alanine transaminase (**ALT**) compared to control (P < 0.05). Cholesterol levels decreased with the normal diet combined with TVE, while TG levels were reduced with the diets combined with TVE and probiotics (P < 0.05). ALP and ALT levels dropped with low-CP × TVE and normal diet × probiotic, respectively (P < 0.05). Low-CP diets and TVE groups showed reduced litter nitrogen (P < 0.05). These results suggest that probiotics and TVE can alleviate the negative effects of low-CP diets on broiler performance. Additionally, probiotics and TVE improve blood biochemistry and litter quality in broiler chickens.

## Introduction

Diet plays a vital role in the growth and development of broiler chickens. The nutrient density of the diet is a key factor that significantly affects both the growth and health, ultimately impacting the economics of broiler production (Gholami, et al., 2024). Crude protein (**CP**) is particularly important and challenging for nutritionists due to its critical role in poultry health and production. To minimize feed costs (Ahmadi-Sefat, et al., 2022) and reduce environmental impact (Liu, et al., 2021; Mi, et al., 2019) in poultry production, ongoing research is essential to develop strategies that lower CP intake without compromising performance or health (Ahmadi-Sefat, et al., 2022).

Numerous studies have been conducted on the use of low-CP diets in poultry. Reducing CP levels in diets improves the nitrogen utilization ratio and reduced nitrogen excretion in poultry (Applegate, et al., 2008). It has demonstrated that decreasing CP from 22.4% to 17.9% resulted in a 19.2% decrease in nitrogen excretion from 1.2 g to 0.97 g per bird per day (Lemme, et al., 2019). Furthermore, reducing undigested protein in the large intestine has been found to restrict the growth of opportunistic pathogens and promote intestinal health (Greenhalgh, et al., 2020). Experiments have shown that reducing CP from 19% to 17% in broiler chickens from 0 to 21 days led to a 29% decrease in nitrogen excretion and a 7% increase in relative nitrogen retention (Alfonso-Avila, et al., 2022).

However, low-CP diets without a compensatory solution, such as adding amino acids, reduced body weight (De Cesare, et al., 2019) and increased feed conversion ratio (**FCR**) in broiler chicken (Hofmann, et al., 2020). Therefore, incorporating probiotics (Bonos, et al., 2021; Shargh, et al., 2012; Zeng, et al., 2021) and medicinal plants (Farahat, et al., 2021; Habibi, et al., 2022) into the diet is strategic approaches that can significantly enhance both the performance and physiological responses of the poultry. Moreover, using probiotics (Krysiak, et al., 2021) and medicinal plants (Ayalew, et al., 2022) to establish a beneficial and healthy microflora in the gastrointestinal tract has been proposed as a viable alternative of antibiotics.

Probiotics, as an alternative to antibiotics, are live bacteria, fungi, or yeasts that support the gastrointestinal flora, which may enhance the growth performance and health status of poultry (Jha, et al., 2020). Probiotics have shown beneficial effects in poultry under certain conditions, though results can vary across studies. Research indicates that probiotics may contribute to gut health by increasing beneficial bacterial populations and reducing potentially harmful pathogens (Khan and Naz, 2013)​. It has been suggested that probiotics can enhance immune responses, nutrient absorption and improve feed conversion ratios, which may contribute to better growth performance in poultry (Jha, et al., 2020). The probiotic used in this experiment was Protexin, a multi-strain probiotic that includes *Lactobacillus Acidophilus, Lactobacillus Rhamnosus, Lactobacillus Bulgaricus, Lactobacillus Plantarium, Bifidobacterium Bifidum, Faecium Enterococcus, Thermophilus Streptococcus, Oryzae Aspergillus*, and *Candida Pintolopessi*. Numerous studies have confirmed the positive effects of this probiotic. For instance, in our pervious study, Protexin has been shown to promote weight gain and improve feed conversion ratios in broiler chickens (Shargh, et al., 2012).

Various medicinal plants and their derivatives have been investigated as alternatives to antibiotics for promoting poultry performance (El-Ashram and Abdelhafez, 2020; Habibi, et al., 2022; Rahmatnejad, et al., 2024; Shargh, et al., 2012). Thyme (Thymus vulgaris) offer significant benefits in poultry nutrition and health. Known for its high nutritive value, thyme is rich in essential nutrients such as vitamins (including vitamin C and vitamin A precursors), minerals (like iron, manganese, and calcium), and antioxidants such as thymol and carvacrol (Iftikhar, et al., 2023). These compounds contribute to improved immune function, digestive health, and overall growth performance in poultry (Attia, et al., 2018; Attia, et al., 2017; Noruzi, et al., 2022). The principal components found in Thyme essential oils include Thymol, Carvacrol, Linalool, Apigenin, Eugenol, p-cymene, γ-terpinene, and Rosmarinic acid (Azaz, et al., 2004; Iftikhar, et al., 2023). These components exhibit antimicrobial properties (Demir, et al., 2005) which can aid in reducing the prevalence of harmful pathogens like *Salmonella* and *E. coli* in poultry. Noruzi, et al. (2022) showed that supplementation of 250 mg/kg thyme essential oil to broiler chickens diets improved feed intake, weight gain, and body weight values. Overall, integrating thyme and its derivatives into poultry diets not only enhances nutritional content but also promotes health and welfare, making it a valuable natural supplement in poultry production.

This study aims to evaluate the effects of Thymus vulgaris extract (**TVE**) and probiotic (Protexin) on the growth performance, blood parameters, intestinal morphology, and litter quality of Arian broiler chickens fed low-protein diets. The primary hypothesis of this study is that lowering dietary CP levels can help reduce litter nitrogen and emissions, thereby enhancing environmental sustainability. Furthermore, it is hypothesized that the inclusion of probiotic and TVE can mitigate the adverse effects of low-CP diets, thus improving the overall performance of broiler chickens.

## Materials and methods

The methods used in this study were approved by the Animal Ethics Committee of the Animal Science Faculty at Gorgan University of Agricultural Sciences and Natural Resources, under protocol #98/181/260 FA.

### Thymus vulgaris extract preparation and bioactive ingredients

Thyme leaves obtained from a traditional plant store were air-dried in a laboratory for five days and ground into fine particles using a simple hammer mill. One hundred grams of this powder were soaked in 900 cc of 80% ethanol for 24 hours. The resulting mixture was homogenized and filtered. The pure solution obtained was then evaporated to produce the hydro-alcoholic extract (Farag, et al., 2024). To identify the bioactive compounds, the samples were sent to the central laboratory for gas chromatography-mass spectrometry (GC-MS) analysis (Table 1).

**Table 1:** Components of thyme vulgaris extract determined by GC-MS (%).

o-Cymene	1.67
Terpinene	1.06
Ethyl2-propan-2-ylcarbonate	1.02
Trans-linalool oxide	1.05
1,6-Octadien-3-ol, 3,7-dimethyl (linalool)	28.54
Terpendiol 1	3.77
Carvacrol methyl ether	1.08
1,3,7-Octatriene, 3,7-dimethyl	2.70
2,6-Dimethyl-1,7-Octadiene-3,6-diol	1.26
Thymol	7.07
Carvacrol	20.22
Succinic acid, isohexyl trans-hex-3-enyl ester	5.18
Caryophyllene	2.40
Tetradecamethylcycloheptasiloxane	2.75
Spathulenol	1.01
Caryophyllene oxide	1.69
Thiophene	4.35
Cyclooctasiloxane	1.28
Phytol	3.42
Other	8.48

### Experimental design and diets

A total of 300 one-day-old male Aryan chicks were obtained from a local commercial hatchery.

On the first day of age, the chicks were weighed and assigned to 30 pens in a completely randomized design with a 2 × 3 factorial arrangement, comprising 6 treatments and 5 replicate groups of 10 birds each. The room temperature was initially set at 32°C for the first 3 days of the trial and was gradually reduced by 3°C per week until it reached 20°C. The relative humidity was maintained at 50 to 60% throughout the experiment. The birds had free access to feed and water during the experimental period.

This research used a 4-phase feeding program that included starter (0–14 d), grower (14–24 d), finisher 1 (25–35 d), and finisher 2 (36–42 d) diets. The experiment consisted of a 2 × 3 factorial arrangement of treatments, including 2 concentrations of CP and three supplementations. The two CP levels were: a control group with 22.00%, 20.00%, 18.50%, and 17.50% in the starter, grower, finisher 1, and finisher 2 phases respectively, and a low-CP diet group with a 5% reduction in CP content, resulting in levels of 20.9%, 19%, 17.57%, and 16.62% in the respective phases. The three supplementations were: no additive, probiotic at 0.1 g/kg of diet, and TVE at 1 ml/L of drinking water. The feed form was mash, and the probiotic was supplemented as a top-dress during the feed formulation process to create respective diets. The feed components and chemical composition of the different experimental diets are shown in Table 2.

**Table 2:** Ingredient and nutrients composition of basal diets.

	Starter (d 1-14)		Grower (d 15-24)		Finisher 1 (d 25-35)		Finisher 2 (d 36-42)	
	Control	Low-CP	Control	Low-CP	Control	Low-CP	Control	Low-CP
**Ingredient, %**								
Corn	55.33	58.17	59.38	62.92	63.18	66.37	66.58	69.55
Soybean meal	36.90	35.40	34.06	31.07	30.50	27.80	27.60	25.10
Corn gluten meal	2.00	1.00	0.00	0.00	0.00	0.00	0.00	0.00
Soybean oil	0.90	0.68	1.80	1.27	2.15	1.65	1.60	1.12
Dicalcium phosphate	1.96	1.92	1.96	1.96	1.51	1.54	1.54	1.56
Limestone	1.08	1.08	0.97	0.98	0.91	0.91	0.92	0.92
Sodium chloride	0.36	0.32	0.36	0.36	0.36	0.36	0.36	0.36
Sodium bicarbonate	0.45	0.45	0.45	0.45	0.45	0.45	0.45	0.45
l-Lysine-HCl	0.20	0.18	0.20	0.18	0.16	0.16	0.17	0.18
dl-Methionine	0.26	0.25	0.26	0.25	0.23	0.21	0.22	0.20
l-Threonine	0.06	0.05	0.06	0.06	0.05	0.05	0.06	0.06
Vitamin and mineral premix	0.5	0.5	0.5	0.5	0.5	0.5	0.5	0.5
Total	100	100	100	100	100	100	100	100
**Nutrient composition (%, unless otherwise stated)**								
ME (kcal/kg)	2870	2870	2950	2950	3025	3025	3025	3024
Crude protein	22.00	20.90	20.00	19.00	18.50	17.57	17.50	16.62
Calcium	0.96	0.96	0.87	0.87	0.78	0.78	0.78	0.78
Available phosphorus	0.48	0.48	0.44	0.44	0.39	0.39	0.39	0.39
Sodium	0.16	0.16	0.16	0.16	0.16	0.16	0.16	0.16
Lysine	1.18	1.12	1.07	1.01	0.98	0.93	0.93	0.88
Methionine	0.58	0.55	0.53	0.50	0.49	0.46	0.47	0.44
Methionine + Cystine	0.88	0.83	0.81	0.76	0.75	0.71	0.72	0.68
Threonine	0.78	0.74	0.72	0.68	0.66	0.62	0.63	0.59
Tryptophan	0.23	0.22	0.21	0.20	0.20	0.19	0.17	0.16
Arginine	1.34	1.27	1.24	1.18	1.15	1.09	1.00	0.95
Histidine	0.53	0.50	0.49	0.47	0.46	0.44	0.42	0.40
Leucine	1.75	1.66	1.54	1.46	1.46	1.39	1.35	1.28
Isoleucine	0.84	0.80	0.76	0.72	0.70	0.67	0.62	0.59
Valine	0.92	0.87	0.83	0.79	0.78	0.74	0.70	0.67
Phenylalanine	1.05	1.00	0.94	0.89	0.87	0.83	0.79	0.75
Glycine	0.73	0.69	0.68	0.65	0.64	0.61	0.56	0.53

Vitamin-mineral premix (per kilogram of diet): 9,000 IU; cholecalciferol, 2,000 IU; dl-α-tocopheryl acetate, 12.5 IU; menadione sodium bisulfite, 1.76 mg; choline chloride, 320 mg; nicotinic acid, 28 mg; calcium d-pantothenate, 6.4 mg; riboflavin, 3.2 mg; pyridoxine, 1.97 mg; thiamine, 1.2 mg; folic acid, 0.38 mg; biotin, 0.12 mg; cyanocobalamin, 0.01 mg; FeSO_4_·7H_2_O, 80 mg; MnSO_4_·H_2_O, 60 mg; ZnO, 51.74 mg; CuSO_4_·5H2O, 8 mg; iodized NaCl, 0.8 mg; Na_2_SeO_3_, 0.2 mg/kg of diet.

The probiotic used in this experiment was Protexin, which contains *Lactobacillus Acidophilus* 2.14 × 10^8^, *Lactobacillus Rhamnosus* 2.28 × 10^8^, *Lactobacillus Bulgaricus* 2.22 × 10^8^, *Lactobacillus Plantarium* 1.28 × 10^8^, *Bifidobacterium Bifidum* 2.10 × 10^8^, *Faecium Enterococcus* 5.60 × 10^8^, *Thermophilus Streptococcus* 4.18 × 10^8^, *Oryzae Aspergillus* 5.60 × 10^7^, and *Candida Pintolopessi* 5.68 × 10^7^.

### Productive performance

Feed intake (**FI**) was determined by calculating the difference between the quantity of feed offered and the amount refused by the birds during the periods of starter, grower, finisher 1, and finisher 2. Body weight gain (**BWG**) was recorded during the mentioned time intervals. FCR was also calculated by dividing the FI by the BWG. Additionally, other production indices, including the protein efficiency ratio (**PER**) as defined by Donadelli, et al. (2019), the energy efficiency ratio (**EER**) according to Shoaib, et al. (2022), and the European production efficiency factor (**EPEF**) according to Biesek, et al. (2022), were calculated using the following formulas:

PER=BWG,g/CPintake,g

EER=BWG,g/energyintake,Kcal

EPEF=100×(livability,%×liveweight,kg/age,d×FCR)

Growth performance was also calculated for the total period of 0 to 42 days.

### Intestinal morphology

At d 42, for the morphological study, 2 cm pieces were cut from the central portions of each jejunum segment, washed with distilled water, and fixed in 10% neutral-buffered formalin. Each intestinal tissue segment was cut into 5mm cross-sections with a microtome, placed on a glass slide, and examined using a light microscope (Olympus CX31, Shinjuku). The morphological measurements of villus height (VH), villus width (VW), and crypt depth (CD) were performed with a light microscope using computer-aided light microscope image analysis. Villus height (VH) was measured from the top of the villus to the upper part of the lamina propria. Crypt depth (CD) was determined from the base upwards to the region of transition between the crypt and villus. Villus width (VW) was measured at the widest area of each villus. Additionally, the villus surface area (VSA) was calculated using the formula (Arak and Karimi Torshizi, 2021):

$$\text{VSA}\left(mm^{2}\right)=2\pi \times \left(\text{VW}/2\right)\times \text{VH}$$

where VW and VH are in micrometers (µm). Reported values are the means of 10 villi from each bird.

### Blood biochemistry and liver enzymes activity

At day 42, blood samples were collected from the left-wing vein of one randomly selected bird per pen following an 8-hour fasting period. The samples were then centrifuged at 3,000 g for 10 minutes at room temperature, and the resulting serum was stored at −20°C until analysis. The serum samples were tested for triglycerides, cholesterol, total protein, alkaline phosphatase (ALP), alanine transaminase (ALT), aspartate transaminase (AST), albumin, and globulin using commercial kits (Pars Azmoon Co., Tehran, Iran) and spectrophotometric methods.

### Litter quality

For litter quality evaluation, on day 42, 10 g of litter was randomly collected from different locations within each pen to ensure a representative sample. The litter samples were then placed in a 100 ml beaker from each individual pen. 50 ml of distilled water was added and stirred with a glass rod. The mixture was left at room temperature for 30 minutes. Subsequently, the pH was measured using a calibrated portable pH meter (HANNA®pHep, pocket pH tester). Litter nitrogen and moisture were determined following the standard protocol of AOAC Inter-National AOAC (2005).

### Statistical analysis

Data were first tested for normality of residual (ShapiroWilk test) and homogeneity of variance (Leven test) considering each pen as the experimental unit. The GLM procedure of SAS 9.4. was used to analyze the data for a 2 × 3 factorial arrangement of treatments in a completely randomized design. The 2 factors were dietary CP level (normal vs low) and supplementation type (no additive, probiotic at 0.1 g/kg of diet, and TVE at 1 ml/L of drinking water). The applied mathematical model was as follows:

$$Y_{\text{ij}}=\mu +A_{i}+B_{j}+{\left(\text{AB}\right)}_{\text{ij}}+e_{\text{ij}}$$

Where, Y_ij_ = observation, µ = overall average, A_i_ = effect of CP level, B_j_ = effect of supplementation type, (AB)_ij_ = interaction effect of i^th^ CP level × j^th^ supplementation type; e_ij_ = error associated with each observation. Data on growth performance metrics were studied on a pen basis, whilst data on intestinal morphology, serum biochemical parametres, and litter quality were analyzed on an individual bird basis. Dietary CP level and supplementation type were investigated as fix factor. Tukey's multiple tests were run to compare the significant differences (*P* < 0.05). The results are provided as mean values with their respective standard errors.

## Results

### Bioactive ingredients of TVE

Components of TVE determined by GC-MS are shown in Table 1. The GC-MS analysis of TVE indicated that the most bioactive compounds were linalool (28.54%), carvacrol (20.22%) and thymol (7.07%).

### Productive performance

Tables 3, 4, and 5 present the effects of treatments on the productive performance of broiler chickens. The effect of dietary CP on the FI and FCR during the grower period was significant, with broiler chickens fed low-CP diets exhibiting higher FI (P = 0.031) and FCR (P = 0.018) compared to those fed normal-CP diets. During the starter period, the inclusion of probiotic and TVE led to increases in FI (P = 0.016) and BWG (P = 0.024), with the probiotic having a greater effect. In contrast, no significant effects (P > 0.05) of additives on FCR during all periods, and on FI, and BWG, during the grower, finisher 1, finisher 2, and entire experimental periods were observed.

**Table 3:** Effect of experimental treatments on the feed intake (FI), body weight gain (BWG), and feed conversion ratio (FCR) of broiler chickens.

	Starter (d 1-14)			Grower (d 15-24)			Finisher 1 (d 25-35)			Finisher 2 (d 36-42)			Total (d 1-42)		
	FI	BWG	FCR	FI	BWG	FCR	FI	BWG	FCR	FI	BWG	FCR	FI	BWG	FCR
**Protein**															
Normal	158.8	149.1	1.06	608.0^b^	450.3	1.35^b^	880.0	633.8	1.39	1201.2	489.1	2.45	2848.0	1722.4	1.65
Low-CP	159.0	136.2	1.16	630.4^a^	408.0	1.55^a^	902.0	560.6	1.63	1290.0	535.0	2.46	2981.4	1639.9	1.82
SEM	4.55	4.12	0.20	12.75	9.84	0.01	23.14	20.18	0.02	65.59	39.14	0.08	63.551	34.46	0.02
P-value	0.1817	0.051	0.237	0.031	0.504	0.018	0.495	0.897	0.498	0.741	0.970	0.707	0.3489	0.946	0.139
Additive															
No	158.8^b^	149.1^b^	1.06	608.0	450.3	1.35	880.0	633.8	1.39	1201.2	489.1	2.45	2848.0	1722.4	1.65
Probiotic	187.4^a^	169.1^a^	1.15	612.8	419.0	1.46	938.0	573.8	1.65	1436.0	606.7	2.49	3174.2	1761.6	1.80
TVE	173.0^ab^	156.6^ab^	1.10	617.8	434.6	1.42	904.0	572.8	1.58	1173.0	504.7	2.34	2867.8	1668.7	1.71
SEM	5.58	5.05	0.24	15.62	12.05	0.02	28.35	24.72	0.03	80.33	47.93	0.10	77.83	42.21	0.02
P-value	0.016	0.024	0.772	0.497	0.494	0.280	0.492	0.962	0.297	23.54	0.579	0.733	0.065	0.263	0.334
**Experimental diets**															
Normal × No	158.8	149.1	1.06	608.0	450.3^ab^	1.35^c^	880.0	633.8	1.39^c^	1201.2	489.1	2.45	2848.0	1722.4	1.65^b^
Normal × Probiotic	187.4	162.1	1.15	612.8	419.0^ab^	1.46^ab^	938.0	573.8	1.65^a^	1436.0	606.7	2.49	3174.2	1761.6	1.80^a^
Normal × TVE	173.0	156.6	1.10	617.8	434.6^ab^	1.42^bc^	904.0	572.8	1.58^ab^	1173.0	504.7	2.34	2867.8	1668.7	1.71^ab^
Low-CP × No	159.0	136.2	1.16	630.4	408.0^b^	1.55^a^	902.0	560.6	1.63^ab^	1290.0	535.0	2.46	2981.4	1639.9	1.82^a^
Low-CP × Probiotic	177.6	161.9	1.09	678.0	459.4^a^	1.47^ab^	934.0	624.1	1.49^b^^c^	1361.8	545.1	2.53	3151.4	1790.5	1.76^ab^
Low-CP × TVE	156.0	133.7	1.17	653.6	464.8^a^	1.40^bc^	954.0	606.8	1.57^ab^	1251.2	526.8	2.44	3014.8	1732.1	1.74^ab^
SEM	7.89	7.14	0.34	22.09	17.05	0.03	40.09	34.96	0.040	113.60	67.79	0.140	110.07	59.69	0.03
P-value	0.557	0.299	0.62	0.618	0.046	0.006	0.798	0.180	0.001	0.727	0.710	0.937	0.696	0.454	0.032

TVE; Thyme vulgaris extract, SEM; standard error of the means.

a-c Means within a column with no common superscript differ significantly (P < 0.05).

**Table 4:** Effect of experimental treatments on protein efficiency ratio (PER) and energy efficiency ratio (EER) of broiler chickens.

	Starter (d 1-14)		Grower (d 15-24)		Finisher 1(d 25-35)		Finisher 2 (d 36-42)		Total (d 1-42)	
	PER	EER	PER	EER	PER	EER	PER	EER	PER	EER
**Protein**										
Normal	4.26	32.71	3.70^a^	25.13^a^	3.89	23.79	2.33	13.48	3.11	20.43
Low-CP	3.91	30.04	3.23^b^	21.90^b^	3.33	20.40	2.36	13.66	2.82	18.57
SEM	0.060	0.530	0.04	0.300	0.050	0.350	0.080	0.470	0.030	0.240
P-value	0.239	0.239	0.017	0.017	0.375	0.375	0.767	0.767	0.125	0.125
**Additive**										
No	4.26	23.71	3.70	25.13	3.89	23.79	2.33	13.48	3.11	20.43
Probiotic	3.93	30.15	3.43	23.26	3.30	20.19	2.36	13.69	2.84	18.69
TVE	4.12	31.60	3.51	23.85	3.42	20.91	2.44	14.12	2.98	19.63
SEM	0.080	0.650	0.050	0.370	0.070	0.430	0.100	0.580	0.040	0.290
P-value	0.784	0.784	0.280	0.280	0.168	0.168	0.771	0.771	0.299	0.299
**Experimental diets**										
Normal × No	4.26	32.71	3.70^a^	25.13^a^	2.89^a^	23.79^a^	2.33	13.48	3.11^a^	20.43^a^
Normal × Probiotic	3.93	30.15	3.43^b^^c^	23.26^b^^c^	3.30^c^	20.19^c^	2.36	13.69	2.84^b^	18.69^b^
Normal × TVE	4.12	31.60	3.51^a^^b^	23.85^a^^b^	3.42^bc^	20.91^b^^c^	2.44	14.12	2.98^a^^b^	19.63^a^^b^
Low-CP × No	3.91	30.04	3.23^c^	21.90^c^	3.33^b^^c^	20.40^b^^c^	2.36	13.67	2.82^b^	18.57^b^
Low-CP × Probiotic	4.15	31.83	3.38^b^^c^	22.96^b^^c^	3.61^a^^b^	22.11^a^^b^	2.27	13.15	2.91^b^	19.16^b^
Low-CP × TVE	3.89	29.86	3.55^a^^b^	24.10^a^^b^	3.43^b^^c^	21.01^b^^c^	2.39	13.87	2.95^a^^b^	19.38^a^^b^
SEM	0.120	0.920	0.070	0.520	0.100	0.610	0.140	0.820	0.060	0.420
P-value	0.064	0.064	0.006	0.006	0.0009	0.0009	0.905	0.905	0.031	0.031

TVE; Thyme vulgaris extract, SEM; standard error of the means.

a-c Means within a column with no common superscript differ significantly (P < 0.05).

**Table 5:** Effect of experimental treatments on European production efficiency factor (EPEF) of broiler chickens.

	d 14	d 24	d 35	d 42	d 1-42
**Protein**					
Normal	176.00	217.40	272.42	175.09	260.11
Low-CP	149.97	173.64	211.60	169.96	225.73
SEM	4.980	4.480	6.960	8.700	5.910
P-value	0.142	0.181	0.637	0.865	0.493
**Additive**					
No	176.00	217.40	272.42	175.09	260.11
Probiotic	167.32	194.34	218.40	183.68	243.38
TVE	170.73	201.91	226.28	177.68	241.88
SEM	6.100	5.490	8.530	10.660	7.230
P-value	0.413	0.429	0.677	0.834	0.873
**Experimental diets**					
Normal × No	176.00	217.40^a^	272.42^a^	175.09	260.11
Normal × Probiotic	167.32	194.34^b^^c^	218.40^c^	183.68	243.38
Normal × TVE	170.73	201.91^a^^b^	226.28^b^^c^	177.68	241.88
Low-CP × No	149.97	173.64^c^	211.60^c^	169.96	225.73
Low-CP × Probiotic	178.16	204.36^a^^b^	255.12^a^^b^	177.73	253.11
Low-CP × TVE	153.81	209.48^a^^b^	236.29^b^^c^	182.46	249.07
SEM	8.630	7.760	12.060	15.070	10.230
P-value	0.105	0.002	0.001	0.924	0.072

TVE; Thyme vulgaris extract, SEM; standard error of the means.

a-c Means within a column with no common superscript differ significantly (P < 0.05).

Regarding the interaction between dietary CP levels and additives, during the grower period, broiler chickens on low-CP diets without supplementation had significantly lower BWG compared to those supplemented with additives (P = 0.046). Additionally, FCR in low-CP diets without supplementation was significantly worse compared to both normal-CP and low-CP diets supplemented with TVE (P = 0.006). During finisher 1 period (P = 0.001), low-CP diets supplemented with probiotic and during entire experimental period (P = 0.032), low-CP diets supplemented with probiotic and TVE resulted in a comparable FCR to normal-CP diets without additives. There was no significant (P > 0.05) interaction between CP level and additives in some cases (e.g., during the starter and finisher 1 periods). Even though, this indicates that probiotic and TVE mitigate the negative impact of low-CP diets on productive parameters, especially during the grower period.

As shown in Table 4, the main effects of dietary CP levels and types of additives on PER and EER were not significant (P > 0.05). In contrast, CP × additives interactions were observed for PER and EER during the grower, finisher 1, and entire experimental periods (P < 0.05). Low-CP diets supplemented with TVE improved PER and EER to levels comparable to normal-CP diets, indicating that this supplement helps mitigate the negative impact of low-CP diets on productive parameters.

As presented in Table 5, the main effects of dietary CP levels and types of additives on EPEF were not significant (P > 0.05). However, CP × additives interactions were observed at d 24 (P = 0.002) and d 35 (P = 0.001). Broilers fed low-CP diets without any additives showed the worst EPEF, but the inclusion of probiotic in the diet or adding TVE to the water improved EPEF to levels close to those of the control group.

### Intestinal morphology

The effects of treatments on the intestinal morphology of broiler chickens are presented in Table 6. The main effects of dietary CP levels and additives supplementation on the VH, VW, CD, VH: CD, and VSA in the jejunum did not show statistical significance compared to the control group. Also, when considering the interaction effects of CP levels and additives, no significant differences were found in intestinal morphology compared to the control treatment.

**Table 6:** Effect of different levels of protein and additives on intestinal morphology of broiler chickens.

	VH (mµ)	VW (mµ)	CD (mµ)	VSA (mm^2^)
**Protein**				
Normal	1252.0	146.6	232.2	0.57
Low-CP	1268.2	140.6	227.6	0.56
SEM	13.090	7.400	4.110	0.030
P-value	0.555	0.215	0.437	0.305
**Additive**				
No	1252.0	146.6	232.2	0.57
Probiotic	1238.8	163.2	243.0	0.63
TVE	1233.2	168.0	249.2	0.65
SEM	16.030	9.060	5.040	0.030
P-value	0.529	0.440	0.078	0.602
**Experimental diets**				
Normal × No	1252.0	146.6	232.2	0.57
Normal × Probiotic	1238.8	163.2	243.0	0.63
Normal × TVE	1233.2	168.0	249.2	0.65
Low-CP × No	1268.2	140.6	227.6	0.56
Low-CP × Probiotic	1253.8	145.0	239.0	0.57
Low-CP × TVE	1235.2	152.2	244.0	0.59
SEM	22.670	12.820	7.130	0.050
P-value	0.941	0.881	0.996	0.887

TVE; thyme vulgaris extract, SEM; standard error of the means, VH; villus height, VW; villus width, CD: crypt depth, and VSA; villus surface area.

a-c Means within a column with no common superscript differ significantly (P < 0.05).

### Blood biochemistry and liver enzyme activity

The effects of treatments on the blood biochemistry and liver enzyme activity of broiler chickens are presented in Table 7. Low-CP diets significantly reduced albumin level in blood serum (P < 0.05). Additionally, the inclusion of additives resulted in a reduction in blood serum cholesterol (P = 0.006), with TVE having a greater effect. Additives also reduced triglycerides levels (P = 0.002). Moreover, TVE led to lower ALP (P = 0.008), and probiotic resulted in lower ALT (P = 0.009) compared to the control. The interaction of CP levels and additives significantly affected serum cholesterol, triglycerides, ALP, and ALT (P < 0.05). Specifically, the inclusion of probiotic in the diet decreased cholesterol compared to the control group (P = 0.021), and additives reduced triglycerides (P = 0.021). ALP (P = 0.030) and ALT (P = 0.038) were decreased by the combinations of low-CP × TVE and normal diet × probiotic, respectively.

**Table 7:** Effect of experimental treatments on serum biochemical parameters of broiler chickens at d 42.

	Cholesterol	TG	TP	ALP	ALT	AST	Albumin	Globulin
**Protein**								
Normal	156.6	102.8	5.0	1623.0	5.8	242.2	2.4^a^	2.55
Low-CP	159.4	0.98	5.1	1700.2	5.4	240.0	2.2^b^	2.57
SEM	4.65	4.130	0.110	19.46	0.14	6.01	0.04	0.120
P-value	0.201	0.937	0.403	0.402	0.821	0.768	0.031	0.108
**Additive**								
No	156.6^a^	102.8^a^	5.0	1623.0^a^^b^	5.8^a^	242.2	2.4	2.55
Probiotic	139.4^a^^b^	74.6^b^	5.0	1730.6^a^	5.0^b^	239.2	2.2	2.85
TVE	119.6^b^	74.8^b^	4.7	1647.0^b^	5.8^a^	233.6	2.3	2.37
SEM	5.58	5.06	0.14	23.83	0.17	7.36	0.05	0.14
P-value	0.006	0.002	0.172	0.008	0.009	0.910	0.401	0.402
**Experimental diets**								
Normal × No	156.6^a^	102.8^a^	5.0	1623.0^b^	5.8^a^^b^	242.2	2.4	2.55
Normal × Probiotic	139.4^a^^b^	74.6^c^	5.1	1730.6^a^^b^	5.0^c^	239.2	2.2	2.85
Normal × TVE	119.6^b^	74.8^c^	4.7	1647.0^b^^c^	5.8^a^^b^	233.60	2.3	2.37
Low-CP × No	159.4^a^	98.0^a^^b^	5.1	1700.2^a^^b^^c^	5.4^b^^c^	240.0	2.5	2.57
Low-CP × Probiotic	140.8^a^^b^	78.2^b^^c^	4.7	1755.0^b^	5.2^b^^c^	242.2	2.6	2.15
Low-CP × TVE	140.8^a^^b^	74.6^c^	4.6	1615.8^c^	6.1^a^	240.4	2.4	2.20
SEM	7.900	7.150	0.190	33.71	0.020	10.410	0.070	0.200
P-value	0.021	0.021	0.381	0.030	0.038	0.992	0.094	0.221

TVE; Thyme vulgaris extract, SEM; standard error of the means, cholesterol (g/dL), TG; triglycerides (mg/dL), TP; total protein (g/dL), ALP; alkaline phosphatase (U/L), ALT; alanine amino transferase (U/L), AST; aspartate transaminase (U/L), Albumin (g/dL), Globulin (g/dL).

a-d Means within a column with no common superscript differ significantly (P < 0.05).

### Litter quality

The effects of treatments on the litter quality (pH, nitrogen, and moisture) of broiler chickens are presented in Table 8. The main effects of dietary CP levels and types of additives on litter quality were not significant (P > 0.05), except for litter nitrogen percentage, where both low-CP diets (P = 0.047) and TVE groups (P = 0.016) reduced this parameter. The interaction effects of CP levels and additives on litter quality were not significant (P > 0.05).

**Table 8:** Effect of experimental treatments on litter quality of broiler chickens at d 42.

	pH	Nitrogen (%)	Moisture (%)
**Protein**			
Normal	8.64	3.74^a^	31.62
Low-CP	8.61	3.29^b^	31.84
SEM	0.080	0.070	0.360
P-value	0.831	0.047	0.760
Additive			
No	8.64	3.74^a^^b^	31.62
Probiotic	8.71	3.75^a^	31.98
TVE	8.52	3.51^b^	31.24
SEM	0.100	0.080	0.440
P-value	0.840	0.016	0.584
**Experimental diets**			
Normal × No	8.64	3.74	31.62
Normal × Probiotic	8.71	3.75	31.98
Normal × TVE	8.52	3.51	31.24
Low-CP × No	8.61	3.29	31.84
Low-CP × Probiotic	8.59	3.80	31.46
Low-CP × TVE	8.61	3.27	31.06
SEM	0.140	0.120	0.630
P-value	0.751	0.172	0.845

TVE; Thyme vulgaris extract, SEM; standard error of the means.

a-b Means within a column with no common superscript differ significantly (P < 0.05).

## Discussion

GC-MS analysis of TVE showed that the most bioactive compounds were linalool (28.54%), carvacrol (20.22%) and thymol (7.07%). In the study of Gedikoğlu, et al. (2019), GC-MS analysis led to the identification of 25 compounds for Thyme vulgaris, which thymol (55.3% and 50.53%), p-cymene (11.2 and 11.79 %), carvacrol (8.7 and 6.65 %), and β-caryophyllene (4.2 and 4.88 %) are the main components of essential oils when using the hydrodistillation and microwave-assisted extraction methods, respectively. Puvača, et al. (2022) showed that the most bioactive compounds of TVE were thymol (43.20%), carvacrol (16.57%) and p-cymene (18.71%). Previous studies reveal that thyme essential oil contains significant amounts of thymol, carvacrol, β-linalool, and 1,8-cineole, while other components are found only in trace amounts (Abu-Lafi, et al., 2008; Gedikoğlu, et al., 2019; Kowalczyk, et al., 2020; Siroli, et al., 2015). Discrepancies in results can often be attributed to factors such as harvesting time, location, and the type of thyme (e.g., wild), which all affect the yield of essential oils (Abu-Lafi, et al., 2008). Additionally, variations in the method of extraction can lead to differences in the quantity of bioactive components (Gedikoğlu, et al., 2019).

Regarding productive performance (Tables 3, 4, and 5), broiler chickens fed low-CP diets exhibiting higher FI and FCR compared to those fed normal-CP diets. Moreover, in other phases, the reduction in CP levels led to a numerical decrease in FI and a deterioration of the FCR. During all periods except for the finisher 2 phase, a decrease in BWG was also noted. Also, the reduction in dietary CP levels led to a numerical decrease in PER, EER, and EPEF. The results of this experiment, indicating the negative effects of reduced protein levels on broiler chicken performance, are consistent with numerous studies (Ahmadzadeh, et al., 2023; Badawi, et al., 2019; Belloir, et al., 2017; Hernández, et al., 2012). Badawi, et al. (2019) revealed that no marked change in total final body weight, body weight gain and total FI of Cobb 500 broilers fed low-CP diet when compared with the control one. However, in agree with current study, they showed the lowering the dietary CP content by 4% and 6% is associated with a marked increase in FCR compared to control group. Belloir, et al. (2017) showed that reducing the dietary CP content from 19% to 15% did not alter BWG or FI, in contrast to FCR. In agree with our results, they indicated that the FCR was significantly higher in the low- CP diets compared with the control group. In line with present study, also, Hernández, et al. (2012) showed an increase in FCR of male broiler chickens fed low-CP diet. Also, In line with the findings of the current research, Belloir, et al. (2017) demonstrated that a decrease in dietary CP content was associated with a rise in FI. This trend could potentially be explained by the fact that broilers increased their consumption in order to meet their genetic potential for growth, according to the theory of FI and growth proposed by Emmans (1987). However, recent studies have presented results that do not entirely support this hypothesis. Instead of experiencing an increase in FI with reduced dietary CP content, certain broiler genotypes actually decreased their FI (Gous, 2007).

The results for feed intake (FI) during the starter phase align with findings from Rehman, et al. (2020) and (Abdel-Raheem and Abd-Allah, 2011), who reported that probiotic supplementation improved FI. The administration of probiotics facilitated a reduction in gastric emptying duration (Abdel-Raheem and Abd-Allah, 2011) and contributed to the equilibrium of the intestinal microbial community (Hamasalim, 2016), which is essential for the early development of the intestine, both increase FI in the starter phase. However, the observed increases in FI and BWG in this study due to probiotic supplementation are inconsistent with Zhang, et al. (2021), who found no significant effect of 1% probiotics (*Lactobacillus casei, L. acidophilus*, and *Bifidobacterium*) on broiler performance from days 1-21. Our results align with Zhang, et al. (2021) and (Awad, et al., 2009), showing that probiotics did not affect FCR. Although, Zhang, et al. (2021) noted a decrease in FI and an increase in body weight for females, and an increase in FI for males from 1-42 days. This study findings are consistent with other experiments that probiotics have no positive impact on broiler performance (Fujiwara, et al., 2009; Shargh, et al., 2012). Conversely, others have stated the positive effects of probiotics on the growth performance of broilers (Mookiah, et al., 2014; Rehman, et al., 2020; Tarabees, et al., 2019). An improvement impact of probiotic in starter phase in this study on the growth rate may be attributed to enhanced intestinal health and the ability of probiotics to secrete enzymes such as amylase, protease, and lipase, leading to better nutrient digestion and absorption (Bedford, 2000). The observed discrepancies in outcomes may be attributable to a multitude of variables, including birds, probiotic strain, sex, and dose rate.

The main effects of TVE showed numerically increases in FI and BWG of broilers during the starter period, supporting the positive effects of thyme and its derivatives reported by other researchers (Alipour, et al., 2015; Attia, et al., 2017; El-Ghousein and Al-Beitawi, 2009; Mašek, et al., 2014; Najafi and Torki, 2010; Shargh, et al., 2012). As shown in table 1, thymol, carvacrol and linalool are the major components in the TVE, known for their antioxidant, antimicrobial, and digestion-enhancing effects (Bozkurt, et al., 2012; Sethiya, 2016), which may contribute to the numerical increase in BWG during the starter period. No significant effects of TVE on broiler performance specially FCR during all periods, which aligns with our previous study (Shargh, et al., 2012) indicating no marked change in FCR with 1000 ppm thyme in water. Similarly, another study found that thyme extract at 0.2%, 0.4%, and 0.6% did not affect BWG, FI, or FCR of broiler chickens from 1–42 days of age (Pourmahmoud, et al., 2013). The lack of a positive effect of TVE on broiler performance after the starter period contrasts with findings from some studies (Feizi, et al., 2013; Mašek, et al., 2014).

The absence of significant effects on overall growth performance may be related to the nutritional composition of the basal diet and/or environmental conditions. Growth-promoting additives generally have a more pronounced impact when raising condition is suboptimal or diets deficient in essential nutrients or less digestible (Attia, et al., 2016; Mašek, et al., 2014; Pourmahmoud, et al., 2013). Additionally, as previously mentioned, harvesting time, location, and the type of thyme (Abu-Lafi, et al., 2008), and variations in the extraction method can result in differences in the quantity of bioactive components (Gedikoğlu, et al., 2019), which may account for the discrepancies in the results.

The most important finding of the current study was the notable interaction between dietary CP and additives on BWG, FCR, PER, EER, and EPEF. Broiler chickens fed low-CP diets without supplementation showed significantly lower growth performance compared to those receiving additives. However, low-CP diets supplemented with probiotic and TVE resulted in a comparable FCR to normal-CP diets. This indicates that the utilization of an additional probiotic or TVE is advantageous for enhancing the energy and protein utilization in broiler rations with a low-CP concentration, thus playing a crucial role in enhancing productive output. There is now a great deal of interest in the use of low-CP diet (Bregendahl, et al., 2002; El-Faham, et al., 2019; Lemme, et al., 2019; Mirhosseini, et al., 2024) and in developing strategies to enhance nutrient utilization in broiler diets. (Abou-Elkhair, et al., 2020; Aletor, et al., 2000; Attia, et al., 2021; Cowieson, et al., 2020; Dang, et al., 2021; Dean, et al., 2006; Elsayed, et al., 2021; Imari, et al., 2023; Maqsood, et al., 2022; Star, et al., 2021; Wang, et al., 2022). There is limited information on the interaction between the additives used in this study and reduced dietary protein levels. However, as previously mentioned, growth-promoting additives such as probiotics and thyme generally show more pronounced positive effects under suboptimal rearing conditions or when the diet is deficient in essential nutrients.

The current study found that neither the main effects nor the interaction effects of dietary CP levels and additives supplementation significantly impacted the VH, VW, CD, VH: CD, and VSA in the jejunum compared to the control group. Several studies have explored the effects of dietary CP levels on intestinal morphology in broilers, with varying results. For instance, Abbasi, et al. (2014) reported no significant changes in VH: CD ratio in broilers fed low-CP diets, which is consistent with the findings of the present study. However, Abbasi, et al. (2014) did observe a reduction in VH of jejunum when dietary CP was reduced to 90% of recommended levels. This reduction was attributed to the role of proteins in maintaining intestinal viability and mass, as well as providing energy for normal intestinal functions. Gastrointestinal tissues are known for their high protein turnover rates, and high-protein diets supply adequate nutrients, particularly CP, for basal metabolism and the development of intestinal structure. In contrast to our findings, Ding, et al. (2016) demonstrated that lowering dietary CP levels led to significant reductions in both VH and the VH: CD ratio in the duodenum, jejunum, and ileum. Their hypothesis suggests that suboptimal protein quality and concentration can negatively affect intestinal development and function, as supported by Wijtten, et al. (2010).

In terms of additive effects, probiotics are often considered to influence intestinal wall thickness by reducing harmful bacteria (Olnood, et al., 2015). A longer small intestine typically indicates a larger digestive and absorptive area, which could enhance nutrient utilization and absorption, thereby improving growth performance. While this study did not observe improvements in the intestinal morphology, the probiotics used may still benefit intestinal development through other mechanisms, such as increasing the total small intestine length, as suggested by Olnood, et al. (2015). Song, et al. (2014) indicated that supplementing broiler diets with a probiotic mixture did not affect CD and VH:CD ratio of broiler chicken, which is consistent with our findings. Similarly, other studies have shown that probiotic supplementation did not influence VH, CD and VH:CD ratio compared to control treatments at different ages (Olnood, et al., 2015; Wang, et al., 2016).

There were no significant effects of TVE on intestinal morphology, consistent with our previous study (Shargh, et al., 2012) indicating no marked change in VH, CD and VH:CD ratio with 1000 ppm thyme in water. Similarly, García, et al. (2007) observed no significant impact on intestinal morphology in broiler chickens at 42 days of age when using 200 ppm of a plant extract blend containing oregano, cinnamon, and pepper essential oils, or 5,000 ppm of hydroalcoholic plant extract from sage, thyme, and rosemary leaves, which aligns with the findings of the current study.

Low-CP diets significantly reduced serum albumin, which supported by the findings of Hernández, et al. (2012) indicating a 3% reduction in dietary protein content reduced plasma albumin in broiler chickens during the prestarter, starter, and finisher phases. The decrease in albumin could be attributed to a deficiency in crude protein and, consequently, the amino acids available to the birds (Hernández, et al., 2012).

There is a notable decrease in serum cholesterol, triglycerides, and ALT in birds fed diet supplemented with probiotic. Gong, et al. (2018), also observed a significant reduction in serum total cholesterol and triglycerides in broilers receiving probiotics, aligning with our findings. Similarly, Mushtaq, et al. (2024) reported improved liver function in broilers fed probiotic-supplemented diets, likely due to the antioxidant properties of probiotics, which neutralize free radicals produced during feed metabolism in the liver, preventing hepatic cellular damage (Ahmad, et al., 2024; Ognik, et al., 2017). Feed is a major source of liver toxicity, particularly from aflatoxins, and probiotics assist in detoxifying, thereby normalizing liver function and improving liver biomarkers (Śliżewska, et al., 2019).

The inclusion of TVE led to a reduction in serum cholesterol, triglycerides, and ALP levels. This aligns with previous studies, which reported significant decreases in triglycerides (Pourmahmoud, et al., 2013) in broilers supplemented with thyme extract. However, (Demir, et al., 2003) found that a powder mixture of medicinal plants (garlic, thyme, cinnamon, and oregano) did not significantly affect plasma triglyceride concentrations in broilers, which contrasts with our results. The reduction in ALP levels with TVE supplementation is consistent with Attia, et al. (2018), who demonstrated the beneficial effects of thyme extract on liver enzyme activity in broilers reared under hot climate. The decreased ALP in the TVE-supplemented groups suggests an overall improvement in health and enhanced liver function due to TVE, which is known to be a good source of PUFA and antioxidants (Attia, et al., 2018).

The main effects of dietary CP levels and types of additives showed litter nitrogen percentage reduced in birds fed low-CP diets and TVE group. The interaction effects of CP levels and additives on litter quality were not significant. Consistent with current findings, a meta-analysis study showed the potential to reduce nitrogen excretion (Alfonso-Avila, et al., 2022). They noted that lowering the dietary CP content from 19% to 17% is associated with a 29% reduction of N excretion, and a 2.2% decrease in litter moisture in broilers aged 0–21 d, which the latter is disagree with our results. Reducing the dietary CP content decreased the nitrogen content of the manure, which also confirms previous reports on manure and fresh droppings (Belloir, et al., 2017; Hernández, et al., 2012; Lemme, et al., 2019; Si, et al., 2004). However, these studies showed decreased the moisture content of the manure by low-CP diets which disagreed with the current findings. The lower litter nitrogen in birds receiving a low-CP diet may be attributed to an enhancement in nitrogen retention, thereby indicating an appropriate nitrogen balance in the diet (Bregendahl, et al., 2002). A plausible explanation for the variations observed in litter moisture across the results may be attributed to the protein content of the diets employed in the respective experiments. In line with our results, Mohammed, et al. (2021) showed no significant differences in the litter moisture and pH of broiler chickens fed diets supplemented with a probiotic, *Bacillus subtilis*. Also, disagree with our results, Such, et al. (2023) indicated that using a symbiotic additive containing *Bacillus subtilis*, inulin, and *Saccharomices cerevisiae* reduced urinary and faecal nitrogen composition of the broiler chickens excreta. Such, et al. (2023) suggest that components of the symbiotic treatment enhanced the conversion of ammonia to bacterial protein in the cecum, which may have contributed to a reduction in urinary nitrogen. Probiotic bacteria can modify protein digestibility and the proteolytic breakdown of the non-digested protein in the hind gut segments (Stanley, et al., 2015), resulting in lower litter nitrogen. Such, et al. (2023) suggest that the components of the symbiotic treatment increased the conversion of ammonia to bacterial protein in the cecum. This may have led to a decreased urinary nitrogen. Moreover, it has been reported that incorporating ingredients like antibiotics, prebiotics, probiotics, and symbiotics can reduce litter ammonia formation by decreasing bacterial activity in the digestive tract (Mahardhika et al., 2019). Comparing these results with studies using other probiotic strains is not particularly informative, as birds' responses to probiotics can vary depending on the dosage and specific strains used. There remains limited information on the effects of TVE on litter quality. However, the findings from previous research (Chowdhury, et al., 2018; Park and Kim, 2020) indicate that dietary supplementation with phytogenic feed additives can enhance nitrogen digestibility in broilers and decreasing nitrogen excretion to the environment, resulting in reduced nitrogen levels in both excreta and litter.

## Conclusions

The findings suggest that a 5% reduction in dietary CP negatively affects the productive performance of broiler chickens. However, lowering dietary CP levels can help reduce litter nitrogen and emissions, thus enhancing environmental sustainability. It can be concluded that the inclusion of probiotic and TVE can ameliorate the detrimental effects of low-CP diets on the growth performance of broiler chickens, while also improving blood biochemistry.
